# Anti-apoptotic potential of several antidiabetic medicinal plants of the eastern James Bay Cree pharmacopeia in cultured kidney cells

**DOI:** 10.1186/s12906-018-2104-1

**Published:** 2018-01-30

**Authors:** Shilin Li, Sarah Pasquin, Hoda M. Eid, Jean-François Gauchat, Ammar Saleem, Pierre S. Haddad

**Affiliations:** 1 Department of Pharmacology and Physiology, Université de Montréal, P.O. Box 6128, Downtown Postal Station, Montreal, (Quebec) H3C 3J7 Canada; 2 Natural Health Products and Metabolic Diseases Laboratory, CIHR Team in Aboriginal Antidiabetic Medicines and Montreal Diabetes Research Center, CRCHUM, Montreal, Canada; 3 Department of Pharmacognosy, Faculty of Pharmacy, Beni-Suef University, Beni-Suef, 62514 Egypt; 4 Center for Advanced Research in Environmental Genomics, University of Ottawa, Ottawa, K1N 6N5 Canada

**Keywords:** Aboriginal traditional medicine, Diabetic nephropathy, MDCK cells, Annexin V, Propidium iodide, Caspase

## Abstract

**Background:**

Our team has identified 17 Boreal forest species from the traditional pharmacopeia of the Eastern James Bay Cree that presented promising in vitro and in vivo biological activities in the context of type 2 diabetes (T2D).

We now screened the 17 plants extracts for potential anti-apoptotic activity in cultured kidney cells and investigated the underlying mechanisms.

**Methods:**

MDCK (Madin-Darnby Canine Kidney) cell damage was induced by hypertonic medium (700 mOsm/L) in the presence or absence of maximal nontoxic concentrations of each of the 17 plant extracts. After 18 h’ treatment, cells were stained with Annexin V (AnnV) and Propidium iodide (PI) and subjected to flow cytometry to assess the cytoprotective (AnnV^−^/PI^−^) and anti-apoptotic (AnnV^+^/PI^−^) potential of the 17 plant extracts. We then selected a representative subset of species (most cytoprotective, moderately so or neutral) to measure the activity of caspases 3, 8 and 9.

**Results:**

*Gaultheria hispidula* and *Abies balsamea* are amongst the most powerful cytoprotective and anti-apoptotic plants and appear to exert their modulatory effect primarily by inhibiting caspase 9 in the mitochondrial apoptotic signaling pathway.

**Conclusion:**

We conclude that several Cree antidiabetic plants exert anti-apoptotic activity that may be relevant in the context of diabetic nephropathy (DN) that affects a significant proportion of Cree diabetics.

## Background

Diabetes mellitus (DM), first officially recorded in ancient Egypt, was initially considered as a rare condition in which patient urinated excessively and lost weight [1]. Clinically, DM refers to a group of metabolic diseases in which there is a chronic hyperglycemic condition as a result of defects in insulin secretion, insulin action or both [2, 3]. Due to compromised insulin secretion or sensitivity, postprandial metabolism is disturbed, resulting in less efficient uptake and utilization of glucose by the targeted tissues, further leading to elevated blood glucose level, more utilization of proteins and fats [4].

The prevalence of DM is rapidly increasing worldwide as a result of sedentary lifestyle, obesity, ageing, smoking, high blood pressure, genetic predisposition, psychiatric disorder such as depression and so on [5]. DM is predicted to affect 366 million individuals by the year 2030 [5–8]. Notably, Aboriginal populations worldwide are disproportionally affected by T2D. This has been linked to rapid environmental changes and to potential genetic predisposition to higher conservation of food calories [9–13]. For instance, the age-adjusted prevalence of T2D in the Cree populations of Eeyou Istchee (CEI - Eastern James Bay area of Quebec, Canada) averaged 29% in 2009 [11, 14]. Cree communities also suffer from higher prevalence of diabetic complications, notably DN. This is due at least in part to the cultural inappropriateness of modern drug treatments [15, 16].

In western countries, end-stage renal disease (ESRD) is mainly caused by DN [17]. Development of proteinuria, considered as the key feature of DN, is related to the decline of glomerular filtration rate (GFR), eventually leading to a progressive loss of renal function [18]. Hypertension and poor glycaemic control are usually associated with DN [19, 20]. More importantly and according to numerous recent studies, it is well established that renal tubular cell apoptosis contributes to the development of DN, leading to gradual loss of renal mass [21–23]. In this context, different cell models can be used to study renal cell apoptosis, such as MDCK cells subjected to hypertonic stress [24].

The Canadian Institutes of Health Research Team in Aboriginal Antidiabetic Medicines (CIHR-TAAM) was instated in 2003 in an effort to find culturally relevant complementary and alternative approaches to T2D prevention and management for Canadian Aboriginal diabetics. Seventeen plant species stemming from the James Bay Cree traditional pharmacopeia were identified through ethnobotanical surveys and tested using a comprehensive platform of bioassays and animal models of obesity and diabetes to identify the plants’ capacity to improve glycemic control [11, 25–28]. Cree community members also encouraged us to study the potential of the plants to protect kidney cells, since DN is quite prevalent in the James Bay area [15, 16].

In current study, we therefore sought to determine the activity of the same 17 plant extracts to afford renal protection and, hence, their potential to mitigate DN. To achieve this, we developed a bioassay based on well-known MDCK cells, a kidney cell of distal tubule origin, that we stressed with hypertonic medium to induce apoptosis [24, 29–33]. We then used flow cytometry and staining reagents for apoptosis (AnnV) and necrosis (PI). Finally, we assessed the role of several caspases in order to begin understanding mechanisms underlying the anti-apoptotic activity of certain plant species.

## Methods

### Cell culture

MDCK cells were generously provided by Dr. Josette Noël (Department of Pharmacology and Physiology, Université de Montréal) and grown in Eagle’s Minimum Essential Medium (EMEM) supplemented with 10% fatal bovine serum (FBS) and 0.5% antibiotics (PS: Penicillin 100 U/mL, Streptomycin 100 μg/mL) and equilibrated with 5% CO2 95% air at 37 °C. Upon reaching subconfluence, cells were gently detached using 0.25% trypsin.

### Plant extract preparation

A total of 17 Cree medicinal plant species were the object of the current study (Table 1) and their respective maximal nontoxic concentrations (see below) used in MDCK cell line are listed in Table 2. Each species of the 17 plant samples were collected in CEI territory and prepared from air-dried and ground plant material according to previously published methods [27, 28]. Authorization for plant sample collection was obtained and managed by a comprehensive research agreement convened between the three Canadian universities involved (Université de Montréal, McGill University, University of Ottawa), the participating Cree First Nations and the Cree Board of Health and Social Services of James Bay [34]. Dr. Alain Cuerrier, a seasoned taxonomist, ascertained the botanical identity of the plant species and voucher specimens have been deposited at the herbarium of the Montreal Botanical Garden [11]. The collected plant samples were extracted with a standard 80% aqueous ethanol protocol as previously described [27, 28]. All the plant extracts have been well characterized in terms of their phytochemical content in previous studies from our laboratory and that of others (shown in Table 1) [34–84]. All extracts were freeze-dried overnight using a Super Moudylo freeze-dryer (TheromFisher, Brockville, ON, Canada). They were then stored at 4 **°**C in amber containers in a dessicator, both of which were flushed free of oxygen. In such conditions, extracts maintained a stable phytochemical profile and biological activity for several years, as tested using HPLC analyses with different detection systems (e.g. diode array and mass spectrometer) and using several cell-based bioassays.

**Table 1:** Phytochemical characteristics of 17 Cree plant species

Latin Name of Plant Species	Cree Name	Common Name	Plant Part	Major Constituents with Antidiabetic Potential	Other identified Phytochemicals
1. *Abies balsamea* (L.) Mill.	Inaast	Balsam fir	Inner bark	Dehydroabietic acid	Limonene, Camphene, Trans-Zeatin, Dehydrojuvabione, Juvabione, (+)-Isojuvabiol, Abienol
2. *Alnus incana subsp. rugosa* (Du Roi) R.T. Clausen	Atuuspiih	Speckled alder	Inner bark	Oregonin	Taraxerol, Taraxerone
3. *Gaultheria hispidula* (L.) Muhl.	Piyeumanaan	Creeping snowberry	Leaves	Not determined	P-Coumaric acid, Myricetrin, Taxifolin glycoside, Rutin, Quercetin-3-galactoside, Quercetin-3-glucoside, Catechol
4. *Juniperus communis* L.	Kaakaachuminatuk	Ground Juniper	Berries	Not determined	Afzelechin, Sciadopitysin, Longifolene, Scutellarein 6-xyloside, Bilobetin, 6-Hydroxyluteolin 6-xyloside, Quercetin 3-O-L-rhamnoside, Epiafzelechin, Junionone, Junipercomnoside A, Junipercomnoside B, (+)-Isocupressic acid, Communic acid, (+)-Junenol, (+)-Sugiol, Elliotinol, 1-(1,4-Dimethyl-3-cyclohexen-1-yl) ethanone, Geijerone, Junicedral
5. *Kalmia angustifolia* L.	Uischichipukw	Sheep laurel	Leaves	Not determined	Asebotin, Procyanidin A2, Quercetin glycoside, Myricetin
6. *Larix laricina* Du Roi (K. Koch)	Waatinaakan	Tamarack	Inner bark	Awashishinic acid, 13-epitorulosol, Rhapontigenin, Reaponticin	Laricitrin 3-glucoside, Syringetin 3-glucoside
7. *Lycopodium clavatum* L.	Pastinaakwaakin	Common clubmoss	Whole plant	Not determined	8-beta-Hydroxylycopodine, Alpha-Obscurine, O-Acetylfawcettiine, Beta-Dihydrolycopodine, Beta-Lofoline, Lycodoline
8. *Picea glauca* (Moench) Voss	Minhiikw	White spruce	Needles	Not determined	Astringin, Isorhapontigenin 3-O-beta-D-glucopyranoside, Piceatannol, Isorhapontigenin
9. *Picea mariana* (P. Mill.) BSP	Iinaatikw	Black spruce	Cones	Not determined	Astringin, Isorhapontigenin 3-O-beta-D-glucopyranoside, Piceatannol, Isorhapontigenin
10. *Pinus banksiana* Lamb.	Uschisk	Jack pine	Cones	Not determined	Pinobanksin, Cyanidin 3-O-glucoside, Pinosylvin, Pinosylvin methyl ether, Quercetin 3,3′-diglucoside, Kaempferol 3-O-beta-D-(6″-coumaroyl)-glucopyranoside, Helichrysoside, Peonidin 3-O-beta-D-glucopyranoside, Delphinidin 3-O-beta-D-glucopyranoside, Petunidin 3-O-beta-D-glucopyranoside, Oenin, 13-Epimanoyl oxide, Torulosol
11. *Populus balsamifera* L.	Mash-mitush	Balsam poplar	Inner bark	Salicortin A and B	Acetophenone, (+)-alpha-Bisabolol, 2′,4′,6′-Trihydroxydihydrochalcone, 2′,6′-Dihydroxy-4′-methoxydihydrochalcone, 2′,4′,6′-Trihydroxy-4-methoxydihydrochalcone
12. *Rhododendron groenlandicum* (Oeder) Kron and Judd	Kaachepukw	Labrador tea	Leaves	Catechin and epicatechin	Taxifolin, Procyanidin A, Dihydroquercetin, (2R,3R)-3,5,7,3′,4′,5’-Hexahydroxyflavanone, Pyrocatechuic acid, Grayanotoxin I, Procyanidin B2
13. *Rhododendron tomentosum* (Stokes) Harmaja subsp. *subarcticum* (Harmaja) G. Wallace	Wiisichipukw	Northern Labrador tea	Leaves	Not determined	Taxifolin, Procyanidin A, Dihydroquercetin, (2R,3R)-3,5,7,3′,4′,5’-Hexahydroxyflavanone, Pyrocatechuic acid, Grayanotoxin I, Taxifolin glycoside
14. *Salix planifolia* Pursh	Piyeuwaatikw	Tealeaf willow	Inner bark	Not determined	Amentoflavone, Picein, Myrtillin, Catechin-(2′- > 2′)-taxifolin, Catechin-(4alpha- > 6)-epicatechin-(4beta- > 8)-epicatechin, Epicatechin-(4beta- > 6)-epicatechin-(4beta- > 8)-catechin,
15. *Sarracenia purpurea* L.	Ayikataas	Pitcher plant	Whole plant	Isorhamnetin-3-O –glucoside, Kaempferol-3-O-(6″-caffeoylglucoside), Quercetin-3-O-galactoside, Moroniside	Histamine
16. *Sorbus decora* (Sarg.) C.K. Schneid.	Maskumanaatikw	Showy mountain ash	Inner bark	23,28-dihydroxy-lup- 12-ene-3β -caffeate	Aucuparin
17. *Vaccinium vitis-idaea* L.	Wiishichimanaanh	Mountain cranberry	Berries	Quercetin, Quercetin-3-O -glucoside, Quercetin-3-O –galactoside	Arbutin, Procyanidin A1, (+)-Catechin

**Table 2:** List of investigated plant species and the concentrations of the extracts tested in MDCK cells

Species	Abbreviations	Plant Part	Concentration (μg/mL)
1. *Abies balsamea* (L.) Mill.	*A. balsamea*	Inner bark	25
2. *Alnus incana* subsp*. rugosa* (Du Roi) R.T. Clausen	*A. incana*	Inner bark	100
3. *Gaultheria hispidula* (L.) Muhl.	*G. hispidula*	Leaves	100
4. *Juniperus communis* L.	*J. communis*	Berries	25
5. *Kalmia angustifolia* L.	*K. augustifolia*	Leaves	50
6. *Larix laricina* Du Roi (K.Koch)	*L. laricina*	Inner bark	25
7. *Lycopodium clavatum* L.	*L. clavatum*	Whole plant	100
8. *Picea glauca* (Moench) Voss	*P. glauca*	Needles	150
9. *Picea mariana* (P. Mill.) BSP	*P. mariana*	Cones	5
10. *Pinus banksiana* Lamb.	*P. banksiana*	Cones	10
11. *Populus balsamifera* L.	*P. balsamifera*	Inner bark	100
12. *Rhododendron groenlandicum* (Oeder) Kron and Judd	*R. groenlandicum*	Leaves	50
13. *Rhododendron tomentosum* (Stokes) Harmaja subsp. *subarcticum* (Harmaja) G. Wallace	*R. tomentosum*	Leaves	100
14. *Salix planifolia* Pursh	*S. planifolia*	Inner bark	25
15. *Sarracenia purpurea* L.	*S. purpurea*	Whole plant	100
16. *Sorbus decora* (Sarg.) C.K. Schneid*.*	*S. decora*	Inner bark	25
17. *Vaccinium vitis-idaea* L.	*V. vitis-idaea*	Berries	100

Stock solutions of each of the 17 plant extracts were prepared in DMSO with concentrations ranging from 5 to 200 mg/mL. They were diluted in culture medium to the working concentrations shown in Table 2 (the final concentration of DMSO was 0.1% for all the treatments).

### Determination of maximal nontoxic plant extract concentrations

Before screening the plants, maximal nontoxic concentrations were determined by cytotoxicity test that reflects the level of lactate dehydrogenase (LDH) release (LDH Colorimetric kit; Roche, Mannheim, Germany). Cells were seeded at the density of 1.5 × 10^5^/well on 6-well plates. Medium was refreshed around 20 h later when 70% confluence was reached, followed by the addition of each of 17 plant extracts with a series of concentrations, respectively. The range of concentrations selected was based on our previous experience with the 17 plant extracts in other cell lines [11, 27, 28]. After 18 h’ incubation, medium (contains released LDH) was collected on ice then the adherent cells (contains cellular LDH) were lysed by EMEM containing 1% Triton X-100 for 10 min at 37 **°**C, 5% CO_2_. Subsequently, all samples were transferred to Eppendorf tubes followed by centrifugation at 250×g, 4 **°**C, 10 min. LDH in both medium and lysate buffer were quantified by a coupled enzymatic reaction in which a red formazan product was generated, the absorbance of which was measured at 490 nm. To calculate % cytotoxicity for each plant extracts we used the following equation:

$$ \frac{\mathrm{Released}\ \mathrm{LDH}}{\mathrm{Total}\ \mathrm{LDH}\ \left(\mathrm{Released}\ \mathrm{LDH}+\mathrm{Cellular}\ \mathrm{LDH}\right)}\times 100\% $$

Results were analyzed and used to determine the optimal nontoxic concentration for each extract. Each experiment was repeated three times.

### Hypertonic stress protocol

To screen the 17 plant extracts, cells were seeded in 6-well plates at the density of 1.5 × 10^5^/well. Around 20 h later, 70% subconfluence was reached. Cells were then switched for 18 h to 700 mOsm/L hypertonic (made by addition of 200 mM sodium chloride to EMEM culture medium) or isotonic EMEM medium without FBS [24], which contained or not each of the 17 plant extracts at their respective nontoxic maximum concentration. Z-VAD-FMK, a pan caspase inhibitor, was selected as cytoprotective positive control.

### Flow cytometric analysis of AnnV/PI staining

Apoptosis assays were performed using AnnV-FITC (fluorescein isothiocyanate, BD Bioscience, Mississauga, ON) and PI (Thermo Fischer Scientific). 5 mL polystyrene tubes (BD Bioscience, Mississauga, ON)) placed in a laminar flow hood were labeled and preloaded with 500 μl FBS prior to assay. After 18 h’ treatment, medium was transferred and collected in prepared polystyrene tubes; adherent cells were washed once with 1 mL PBS then harvested with 250 μl 0.25% trypsin followed by another 1 mL PBS wash; all polystyrene tubes were subjected to centrifugation at 250 g, 4 **°**C, 5 min. Subsequently, supernatant was discarded and cell pellets resuspended in 500 μL of ice cold binding buffer (10 mM Hepes pH 7.4, 150 mM NaCl, 5 mM KCl, 1 mM MgCl_2_, 1.8 mM CaCl_2_). Cell suspensions were then mixed with 2 μL of AnnV-FITC (AnnV) and 1 μL of PI. After incubation on ice for 5 min in the dark, healthy and damaged cells were determined and analyzed using a FACSCanto II (BD, Biosciences, Bedford, MA, USA) flow cytometer and FlowJo software. Positive staining was confirmed under fluorescence microscopy. Each experiment was repeated three times. As illustrated in the Results section below, PI^+^ Quadrants Q1 and Q2 respectively represent necrosis and late stage apoptosis/secondary necrosis; Q4 Quadrant represents viability (AnnV^−^/PI^−^); Q3 Quadrant (AnnV^+^/PI^−^) represents early stage apoptosis.

### Flow cytometry analysis of caspase 3, 8, 9

As described in the Results section, we selected a subset of plant extracts to study potential underlying mechanisms of MDCK cell protection. Consequently, caspase 3, 8, 9 assays were performed according to manufacturer’s protocol by using the CaspGLOW™ Fluorescein Active Caspase Staining Kit (eBioscience, San Diego, CA, USA). Briefly, cells were detached as described above for AnnV and PI staining before CaspGLOW™ Fluorescein Active Caspase staining was applied. After centrifugation at 250 g, 4 **°**C, 5 min, 300 μL EMEM containing 1 μL CaspGLOW™ Fluorescein Active Caspase was added to each tube, then transferred in a 37 **°**C incubator with 5% CO_2_ for 45 min. Subsequently, cells were subjected three times to a course of wash and spin-down (centrifugation at 500 g, 5 min) at room temperature. Finally, all tubes were subjected to flow cytometry for analysis of respective caspase staining by FACSCanto II (BD, Biosciences, Bedford, MA, USA) flow cytometer and FlowJo software. This assay utilizes inhibitors specific for cleaved caspase-3, − 8 and − 9, respectively. They are directly conjugated to FITC for the detection system. These reagents are cell permeable, non-toxic and irreversibly bind to the cleaved caspase 3, 8, and 9, respectively. Detection of the labeled cells can be determined by flow cytometry.

### Statistical analysis

Results are presented as mean ± SEM of 3 independent experiments with triplicate for each sample. Statistical comparisons between the experimental groups were analyzed by one-way ANOVA and the Bonferroni test as appropriate in software Prism 6 (GraphPad Software Inc., San Diego, CA, USA). A *P* value below 0.05 was considered statistically significant.

## Results

### Determination of maximal nontoxic plant extract concentrations

MDCK cells were treated with 17 plant extracts at different concentrations, respectively. After an overnight incubation (18 h), LDH release was measured. Maximal nontoxic concentrations were determined and results are listed in Table 2. At concentrations listed, all plant extracts exhibited LDH release values that did not exceed 5–10% beyond values obtained with DMSO alone.

### In vitro screen of renal protective potential of 17 antidiabetic medicinal plant extracts and analysis by flow cytometry

**Fig. 1:**
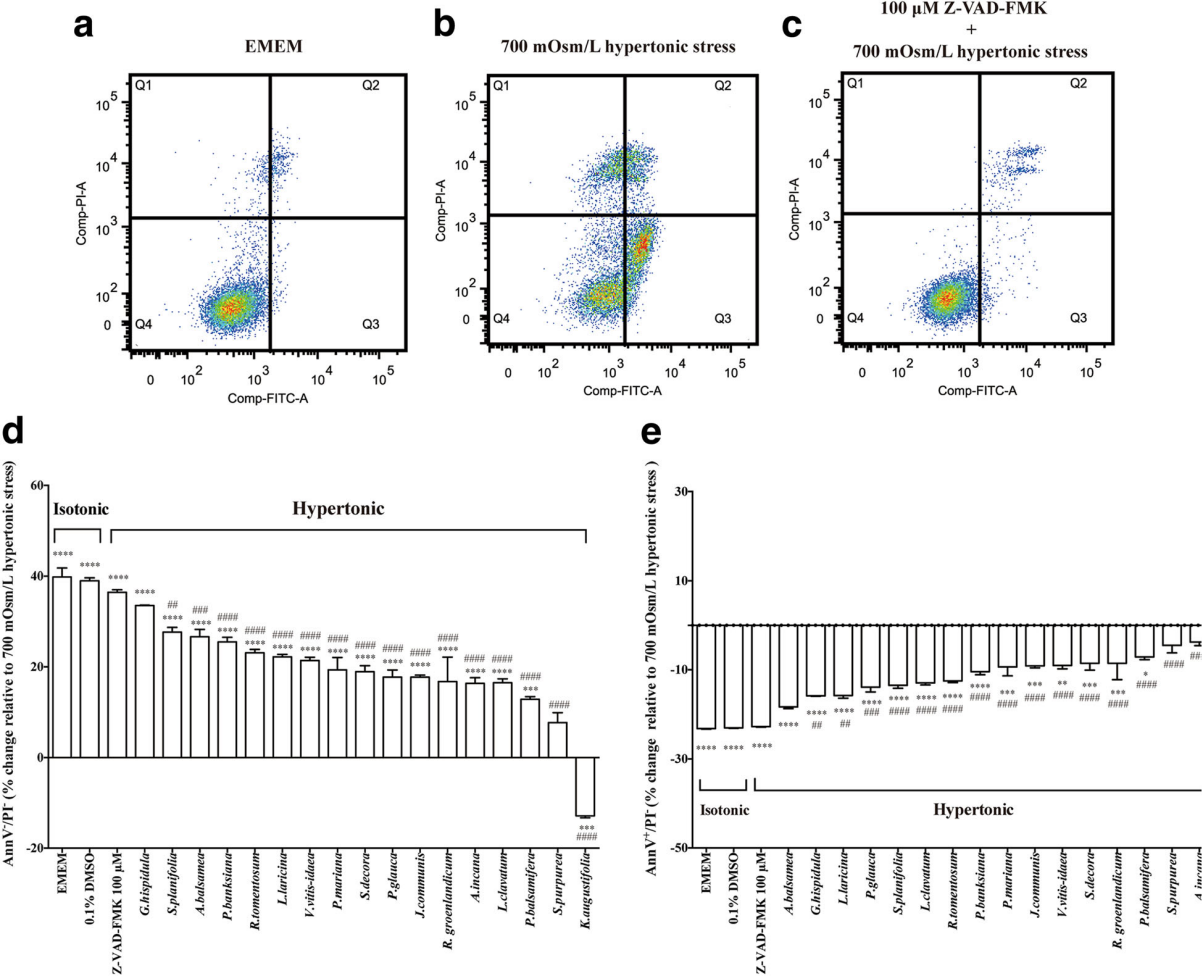
Renal protective potential of 17 antidiabetic medicinal plant extracts. AnnV/PI labeling was performed 18 h after plant extract treatment to assess viability and early stage apoptosis. **a**-**c** Representative pictures of flow cytometry. PI^+^ Quadrants Q1 and Q2 respectively represent necrosis and late stage apoptosis/secondary necrosis; AnnV^−^/PI^−^ Quadrant Q4 represents viable cells; AnnV^+^/PI^−^ Quadrant Q3 represents early stage apoptosis; (**d**) Histogram of change in AnnV^−^/PI^−^ signal (viable cells). Data are presented relative to 700 mOsm/L hypertonic stress to show the improvement in cell viability observed in control (isotonic EMEM), vehicle (0.1% DMSO in isotonic EMEM), positive control (Z-VAD-FMK in hypertonic EMEM) and plant extract treated (in hypertonic EMEM) cells; (**e**) Histogram of change in AnnV^+^/PI^−^ signal (apoptotic cells). Data are presented relative to 700 mOsm/L hypertonic stress to show the reduction in apoptotic cells observed in control (isotonic EMEM), vehicle (0.1% DMSO in isotonic EMEM), positive control (Z-VAD-FMK in hypertonic EMEM) and plant extract treated (in hypertonic EMEM) cells; Results (**d** and **e**) were expressed as means ± SEM for 3 separate experiments. * (*P* < 0.05), **(*P* < 0.01), *** (*P* < 0.001) and **** (*P* < 0.0001) denotes other treatments significantly different from 700 mOsm/L hypertonic stress treatment, one-way ANOVA and Bonferroni test. ## (*P* < 0.01), ### (*P* < 0.001) and #### (*P* < 0.0001) denotes other treatments significantly different from isotonic EMEM, one-way ANOVA and Bonferroni test

In order to identify the respective renal protective and anti-apoptotic potential of the 17 plant extracts in vitro, AnnV/PI double staining was performed. Examined by fluorescent-based flow cytometry and compared to isotonic EMEM (Fig. 1a), 700 mOsm/L hypertonic stress (Fig. 1b) induced substantial PI^+^ (necrosis and late stage apoptosis/secondary necrosis, Q1 and Q2 Quadrants) as well as AnnV^+^/PI^−^ (early stage apoptosis, Q3 Quadrant) signals in MDCK cells. We also used Z-VAD-FMK as a positive cytoprotective control. It is a commonly recognized pan caspase inhibitor, exerting its protective role by irreversibly binding to the catalytic site of caspase proteases [85]. Accordingly, Z-VAD-FMK efficiently protected MDCK cells from the deleterious effects of hypertonic stress (Fig. 1c). In the presence of such 700 mOsm/L hypertonic stress (itself alone resulting in only 53.8% AnnV^−^/PI^−^ staining - viable cells), all plant extracts, except *S. purpurea* and *K. augustifolia*, variably yet significantly improved cell viability as assessed by the change in AnnV^−^/PI^−^ staining (Fig. 1d). Among the 17 plant extracts, *G. hispidula*, *S. planifolia* and *A. balsamea* were most active, respectively increasing AnnV^−^/PI^−^ staining (viable cells) by 33.5%, 27.7% and 26.6%, relative to hypertonic stress alone (*P* < 0.0001, Fig. 1d). In addition, the difference in AnnV^−^/PI^−^ staining observed in the *G. hispidula* group was statistically similar to that seen in the control (isotonic EMEM) and in the positive control Z-VAD-FMK group. On the other hand, *K. augustifolia* was the only plant extract to worsen cell viability under hypertonic stress; in fact, it further reduced AnnV^−^/PI^−^ staining (− 12.9% relative to hypertonic stress, *P* < 0.001, Fig. 1d).

Statistical analysis from Q3 Quadrant (AnnV^+^/PI^−^, Fig. 1e) indicated that hypertonic stress caused a considerable increase of early stage apoptosis (23.5%) compared to isotonic EMEM (0.3%, *P* < 0.0001). *A. balsamea*, *G. hispidula* and *L. laricina* were the most powerful plants protecting against early stage apoptosis, respectively reducing AnnV^+^/PI^−^ staining by 18.4%, 15.9% and 15.8% relative to hypertonic stress alone (*P* < 0.0001, Fig. 1e). Most importantly, *A. balsamea* extract yielded results that were statistically similar to that of the isotonic EMEM group (23.2% reduction in AnnV^+^/PI^−^ staining relative to hypertonic stress, *P* < 0.0001, Fig. 1e) and the Z-VAD-FMK group (22.8% reduction in AnnV^+^/PI^−^ staining relative to hypertonic stress, *P* < 0.0001, Fig. 1e). Again, *K. augustifolia* exhibited its apparent renal toxic action by increasing AnnV^+^/PI^−^ staining relative to hypertonic stress by as much as 25.0% (*P* < 0.0001, Fig. 1e).

We then sought to carry out further analysis to begin understanding potential underlying mechanisms, notably in relation to renal cell apoptosis. Based on the results presented above, we therefore selected a subset of five species. We selected *G. hispidula* and *A. balsamea* as representatives of the best plant species to improve AnnV^−^/PI^−^ staining and reduce AnnV^+^/PI^−^ staining. *R. tomentosum* and *R. groenlandicum* were selected as species that had moderate effects and *S. purpurea* as one of the least effective plant species.

### Mechanism investigation

#### Cleaved caspase 3 activity

**Fig. 2:**
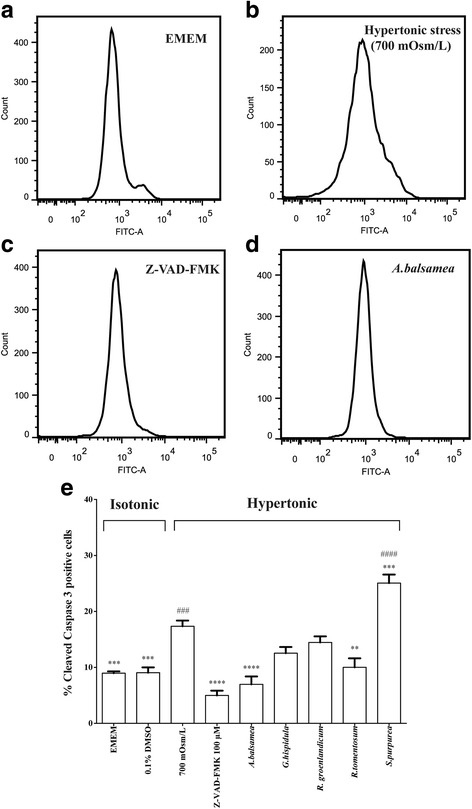
Cleaved caspase 3 activity. Cleaved caspase 3 activity test was performed 18 h after the treatment with respective plant extracts. (**a**-**d**) Representative pictures of flow cytometry. Ordinate indicates cell count whereas abscissa represents fluorescence signal strength. From left to right, 1st peak indicates procaspase 3 and 2nd peak represents cleaved caspase 3 activity. **e** Histogram of percent cleaved caspase 3 positive cells. The percent cleaved caspase 3 activity was calculated as the area of 2nd peak/total area × 100. Results were expressed as means ± SEM for 3 separate experiments. * (*P* < 0.05), **(*P* < 0.01), *** (*P* < 0.001) and **** (*P* < 0.0001) denotes other treatments significantly different from 700 mOsm/L hypertonic stress treatment, one-way ANOVA and Bonferroni test. ## (*P* < 0.01), ### (*P* < 0.001) and #### (*P* < 0.0001) denotes treatment groups significantly different from isotonic EMEM, one-way ANOVA and Bonferroni test

The activity of cleaved (active) caspase 3 following various treatments was then assessed by flow cytometry analysis (Fig. 2, panels a-d). The proportion of cells exhibiting staining for active caspase 3, a hallmark of apoptosis, was significantly increased in the hypertonic stress treated group (Fig. 2b). In comparison, the isotonic EMEM group (Fig. 2a) had a proportion of caspase 3 positive cells that was 48.3% lower than hypertonic stress (*P* < 0.01, Fig. 2a–e). As a positive control, pan caspase inhibitor Z-VAD-FMK largely suppressed the hypertonically induced activity of cleaved caspase 3 (71.2% lower than hypertonic stress, *P* < 0.001, Fig. 2c and e), this effect reaching levels even lower than the isotonic EMEM group (Fig. 2e). Among the representative plant species selected from our primarily screen, a statistically significant reduction in activity of cleaved caspase 3 was observed for *A. balsamea* (Fig. 2d and e) and *R. tomentosum* (59.8% and 42.3% lower than hypertonic stress, respectively, *P* < 0.01, Fig. 2e). *G. hispidula* and *R. groenlandicum* afforded weak protection against cleaved caspase 3 activation induced by hypertonic stress; their caspase 3 activity lying between isotonic EMEM and hypertonic stress group (N.S. versus either group, Fig. 2e). In contrast, *S. purpurea* exhibited more cleaved caspase 3 activity compared to hypertonic stress treated group (44.5% higher than hypertonic stress, *P* < 0.001, Fig. 2e). Aside from *R. tomentosum*, these results are in accordance with the rank order of cytoprotection observed in our primarily screen.

#### Cleaved caspase 8 activity

**Fig. 3:**
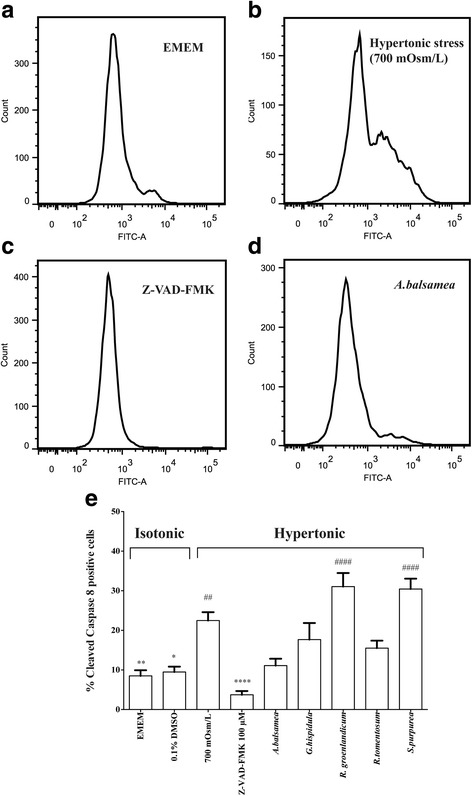
Cleaved caspase 8 activity. Cleaved caspase 8 activity test was performed 18 h after the treatment with respective plant extracts. **a**-**d** Representative pictures of flow cytometry. Ordinate indicates cell count whereas abscissa represents fluorescence signal strength. From left to right, 1st peak indicates procaspase 8 and 2nd peak represents cleaved caspase 8 activity. **e** Histogram of percent cleaved caspase 8 positive cells. The percent cleaved caspase 8 activity was calculated as the area of 2nd peak/total area × 100. Results were expressed as means ± SEM for 3 separate experiments. * (*P* < 0.05), **(*P* < 0.01), *** (*P* < 0.001) and **** (*P* < 0.0001) denotes other treatments significantly different from 700 mOsm/L hypertonic stress treatment, one-way ANOVA and Bonferroni test. ## (*P* < 0.01), ### (*P* < 0.001) and #### (*P* < 0.0001) denotes treatment groups significantly different from isotonic EMEM, one-way ANOVA and Bonferroni test

Secondly, the activity of cleaved caspase 8, which indicates a death receptor signaling pathway, was also assessed (Fig. 3, panels a-d). After MDCK cells were stressed by hypertonic medium (700 mOsm/L) for 18 h, the level of cleaved caspase 8 became significantly higher (22.5%, Fig. 3b) compared to that of the isotonic EMEM group (8.5%, P < 0.01, Fig. 3a). The pan-caspase inhibitor Z-VAD-FMK (Fig. 3, panels c and e) significantly reduced the proportion of cleaved caspase 8 positive cells to 3.7%, slightly lower than isotonic EMEM values (Fig. 3, panels a and e). Except for Z-VAD-FMK, the rest of the selected plant species seemed ineffective at decreasing the proportion of cleaved caspase 8 in a statistically significant manner. In fact, *S. purpurea* and *R. groenlandicum* enhanced the proportion of cleaved caspase 8 positive cells (30.5% and 31.1%, respectively, *P* < 0.0001 Fig. 3e).

#### Cleaved caspase 9 activity

**Fig. 4:**
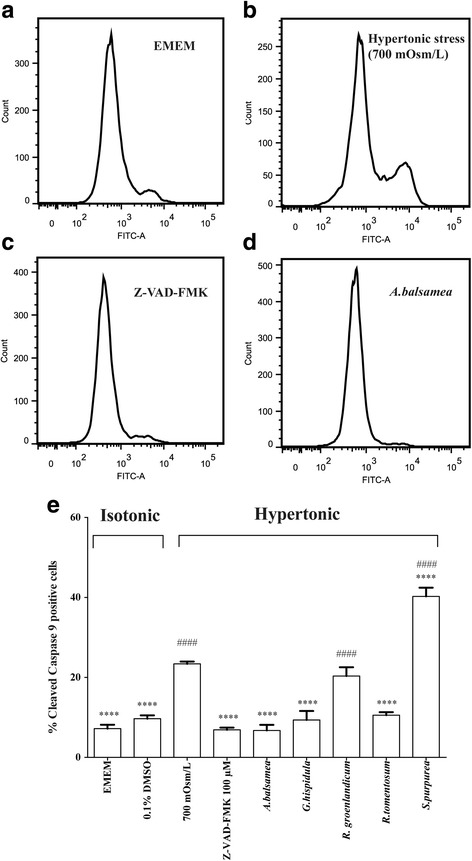
Cleaved caspase 9 activity. Cleaved caspase 9 activity test was performed 18 h after the treatment with respective plant extracts. **a**-**d** Representative pictures of flow cytometry. Ordinate indicates cell count whereas abscissa represents fluorescence signal strength. From left to right, 1st peak indicates procaspase 9 and 2nd peak represents cleaved caspase 9 activity. **e** Histogram of percent cleaved caspase 9 positive cells. The percent cleaved caspase 9 activity was calculated as the area of 2nd peak/total area × 100. Results were expressed as means ± SEM for 3 separate experiments. * (*P* < 0.05), **(*P* < 0.01), *** (*P* < 0.001) and **** (*P* < 0.0001) denotes other treatments significantly different from 700 mOsm/L hypertonic stress treatment, one-way ANOVA and Bonferroni test. ## (*P* < 0.01), ### (*P* < 0.001) and #### (*P* < 0.0001) denotes treatment groups significantly different from isotonic EMEM, one-way ANOVA and Bonferroni test

We finally analyzed the activity of cleaved caspase 9, indicative of the mitochondrial apoptotic pathway, using fluorescent staining and flow cytometry (Fig. 4, panels a-d). Compared to the value of 7.2% obtained in the isotonic EMEM group (Fig. 4, panels a and e), hypertonic stress enhanced the proportion of cleaved caspase 9 positive cells to 23.4% (*P* < 0.0001, Fig. 4, panels b and e). As expected the positive cytoprotective control Z-VAD-FMK reduced the effect of hypertonic stress on caspase 9 positive cells (6.9%, *P* < 0.0001 compared to hypertonic stress group, Fig. 4, panels c and e). In comparison, the effective Cree plant treatments were *A. balsamea*, *G. hispidula* and *R. tomentosum*, with corresponding percentage of cleaved caspase 9 at 6.8%, 9.4% and 10.6%, respectively (*P* < 0.0001 compared to hypertonic stress, Fig. 4, panels d and e). *R. groenlandicum* and *S. purpurea* were again not only ineffective at suppressing the production of cleaved caspase 9 but *S. purpurea* also significantly increased its expression (*P* < 0.0001, Fig. 4e).

## Discussion

The aim of the current study was to begin evaluating the nephroprotective capacity of 17 Cree medicinal plants that were identified as having significant antidiabetic potential in several bioassays related to glucose and lipid homeostasis as well as in animal models of obesity and diabetes [26, 86]. As mentioned, our CEI partners highlighted the fact that their population was suffering disproportionately from DN [34, 87] and wanted us to look into renal protection stemming from their traditional medicinal plants.

We therefore selected the MDCK cell line, which is a very well characterized renal tubular cell model that can serve to assess cytoprotection against various insults [24, 29–33]. We also selected hypertonic stress to cause cell death, notably involving apoptosis [24]. In preliminary experiments, we challenged MDCK cells with different medium osmolarities (including 300, 400, 500, 600, 700 and 800 mOsm/L hypertonic medium, data not illustrated) and found that 700 mOsm/L was the ideal concentration to induce substantial apoptosis, which could be used to test the anti-apoptotic potential of the 17 plant species. This also corresponded with the concentration used successfully by other researchers in the same cell line [24]. This is pertinent in the context of the present studies, since renal tubular apoptosis is recognized as a major contributor for the development of DN [21–23]. Our results with AnnV/PI double staining and flow cytometry clearly demonstrate that a 700 mOsm/L hypertonic stress resulted in significant increases in cell death (PI^+^ quadrants Q1 and Q2; respectively representing necrosis and late apoptosis). Hypertonic stress also significantly enhanced the proportion of cells with AnnV^+^/PI^−^ staining, indicative of early apoptotic cell damage.

Also, importantly, we chose the pan caspase inhibitor Z-VAD-FMK as a positive cytoprotective control introduced in the hypertonic medium. It was very efficient in returning the pattern of AnnV/PI staining to that seen in isotonic EMEM (no hypertonic stress). Thus, our MDCK cells provide an adequate model in which to screen for potential nephroprotective and anti-apoptotic activities of Cree antidiabetic medicinal plants.

When MDCK cells were treated with the various antidiabetic Cree plants, we obtained a wide range of effects; a number of plant extracts almost completely prevented the deleterious effects of hypertonic stress, several were moderately cytoprotective, a few were weakly so and one plant actually enhanced nephrotoxicity, namely *K. angustifolia*. Notably, *G. hispidula* and *A. balsamea* plant extracts were amongst the best performers in maintaining high viability and suppressing apoptosis, being as powerful as Z-VAD-FMK. As mentioned, Z-VAD-FMK is a pan-caspase inhibitor that acts against apoptosis by irreversibly binding to the catalytic site of caspase proteases. As also discussed, apoptosis is involved in cell damage caused by hypertonic stress and is implicated in DN [21–24]. We therefore sought to determine the potential role that caspases could play in the variable cytoprotective activity that we observed for the Cree plants.

Caspases (cysteine-aspartic proteases or cysteine-dependent aspartate-directed proteases) belong to a family called cysteine proteases. They play an essential role in cell apoptosis or programmed cell death and have thus been termed “executioner” proteins [88, 89]. Caspases are regulated at a post-translational level, which allows them to be activated rapidly. Caspases are synthesized as inactive preforms and are cleaved next to aspartate residues upon activation [90]. There are so-called initiator and effector caspases. Initiator caspases possessed specific domains not encountered in effector caspases, such as caspase activation and recruitment domains (CARDs) (e.g., caspase-2 and caspase-9) or a death effector domain (DED) (caspase-8 and caspase-10). These ensure that the caspases can interact with other molecules that regulate their activation. These regulating molecules receive signals from extracellular and intracellular stimuli and interact with initiator caspases, causing their clustering. Such clustering allows initiator caspases to auto-activate and to proceed to activate effector caspases, eventually leading to the amplification of caspase activity through a protease cascade [90], considered as a positive feedback [91]. As mentioned, the activation of caspases can be initiated from extracellular stimuli involving death receptors on the plasma membrane (receptor pathway) or through intracellular stimuli centered in mitochondria (mitochondrial pathway) [92].

Death receptor stimulation activates procaspase-8, whereas the mitochondrial pathway involves the release of cytochrome c and other factors that activate procaspase-9 [24, 92]. Both signaling pathways will eventually lead to downstream activation of caspase-3, which is a major effector in the caspase cascade. Since caspase-3 serves as a convergence point for different signaling pathways, it is therefore well suited as a read-out in an apoptosis assay. In the current studies, we used fluorescent substrates of these three major caspases to probe their activation by flow cytometry. Our results clearly show an increased activity of cleaved caspase 3, 8 and 9 activities in MDCK cells subjected to a 700 mOsm/L hypertonic stress for 18 h. Moreover, the proportion of caspase 8 and 9 “positive” cells was 22.5% and 23.4%, respectively, which indicates that the death receptor and mitochondrial pathways were similarly activated in cells challenged by hypertonic medium.

When we studied the five plant species that were selected to represent strong, medium and low cytoprotective potential in AnnV/PI assays, their rank order of inhibition of hypertonic stress-induced cell damage was mostly maintained for cleaved caspases 3, 8 and 9, except for *G. hispidula* that was ineffective at reducing the activity of caspases 3 and 8, and *R. tomentosum* that was unexpectedly effective in reducing the activity of caspases 3 and 9.

Rather limited knowledge is available regarding *G. hispidula*. In previous studies from our group, the plant extract exerted some cytoprotective potential in preneuronal cells subjected to hyperglycemic stress [27] and moderately stimulated AMPK in cultured hepatocytes [11]. The current studies uncovered a strong cytoprotective potential for renal tubular cells as observed through improved viability (annV^−^/PI^−^ staining) and reduced early stage apoptosis (annV^+^/PI^−^ staining). However, as mentioned *G. hispidula* did not succeed in significantly modulating the activities of caspases 3 and 8 in hypertonically stressed MDCK cells. This suggests that the nephroprotective activity of the plant extract may occur principally through the mitochondrial pathway (significant reduction of cleaved caspase 9) or through pathways other than the classic apoptotic signaling pathways (death receptor or mitochondrial) [93].

Meanwhile, the non-cytoprotective plant *S. purpurea* actually enhanced the activity of all cleaved caspases in hypertonically stressed MDCK cells. Hence, despite the fact that *S. purpurea* previously exhibited a positive impact on muscle cell glucose uptake [34] and, notably, cytoprotection of pre-neuronal cells (potential benefit in diabetic neuropathy [37]), the plant appears to be potentially more harmful to renal cells. Indeed, Elders have cautioned us that the pitcher plant needed to be used carefully for it is considered very powerful.

Another of the weaker nephroprotective plants in our Ann V/PI assay, namely *R. groenlandicum*, appeared to increase the activation of caspase 8 compared to hypertonic stress whereas it had no apparent effect on cleaved caspase 3 and caspase 9 activities. This result is surprising since Labrador tea (*R. groenlandicum*) was previously observed by our group to exhibit several beneficial antidiabetic activities when tested in both in vitro bioassays [36, 94] and in vivo animal models of obesity and mild diabetes [95, 96]. Such antidiabetic activities would be expected to improve renal function (for instance, through reduction of glycemia and improvement of insulin resistance). In fact, we observed improved micro-albuminuria, reduced fibrosis and diminished expression of Bcl2-modulating factor (Bmf) in renal tissues of diet-induced obese mice treated with *R. groenlandicum* in vivo [95]. Interestingly, in the current studies, *R. tomentosum*, a close relative of *R. groenlandicum* also known as Northern Labrador tea, exhibited significant anti-apoptotic potential that was expressed more potently through the suppression of the mitochondrial apoptotic pathway than that of the death receptor pathway. Further studies will thus be necessary to ascertain the complete impacts of Labrador or Northern Labrador tea consumption on the kidney.

In contrast, *A. balsamea* clearly stands out as one of the most powerful renal protective Cree plants that exhibited important anti-apoptotic activities, especially at the level of caspases 3 and 9. Our previous work with *A. balsamea* extracts demonstrated that they can significantly enhance basal and insulin stimulated glucose uptake in cultured skeletal muscle cells and adipocytes [28]. The plant was also the most powerful of Cree species to mitigate liver cell glucose production mechanisms in vitro through both insulin-dependent and insulin-independent mechanisms [86]. In skeletal muscle cells, *A. balsamea* exerted its action through a mechanism similar to that of metformin, involving the activation of AMPK secondary to metabolic stress induced by the disruption of mitochondrial energy transduction (energy depletion), albeit with only mild effects on cell pH or ATP levels [97]. It is interesting to speculate that *A. balsamea*’s effects on mitochondria can also result in triggering anti-apoptotic events but further studies will be necessary to address this point.

Lastly, it is acknowledged that our study provides a good assessment of the efficacy (maximal effect) of the various plant extracts, used at maximal non-toxic concentrations, but does not provide values of EC50 (improvement of cell viability) or IC50 (reduction of apoptosis and caspase activities) that can offer an indication of the relative potencies of the various plant extracts tested. This approach was selected to be consistent with our numerous previous screening studies with the same 17 plant species, carried out in various bioassays related to diabetes and/or its complications [11, 27, 28, 34, 97]. In addition, EC50 and IC50 values are usually applied to drugs and pure compounds acting on a defined target, generally a receptor, and are thus of limited interpretation when applied to complex mixtures such as crude plant extracts used herein.

## Conclusion

In summary, this study demonstrates that *A. balsamea* and *G. hispidula* exhibit the greatest potential of all Cree antidiabetic plants tested to protect kidney cells against damage induced by tubular apoptosis. *A. balsamea* appears to do so by suppressing apoptosis, most likely through actions on the mitochondrial caspase signaling pathway, whereas *G. hispidula* may exert its renal protective potential through mitochondrial and other pathways. In addition, the data generated in the current study suggest that this bioassay involving MDCK cells and flow cytometry may represent a useful and novel tool to screen for nephroprotective agents.

Finally, these findings may have clinical significance for the mitigation of DN that is affecting CEI communities. Indeed, Cree medicinal plant species may contain phytochemical components that can provide novel pharmacological avenues to improve DN treatment and management. More importantly, Cree traditional plant preparations hold promise as complementary therapies that are culturally adapted and respectful. As such, future clinical studies should be encouraged.

## Abbreviations

AnnV: Annexin V
Bmf: Bcl2-modulating factor
CARDs: Caspase activation and recruitment domains
CEI: Cree populations of Eeyou Istchee
CIHR-TAAM: The Canadian Institutes Health Research Team in Aboriginal Antidiabetic Medicines
DED: Death effector domain
DM: Diabetes mellitus
DN: Diabetes nephropathy
EMEM: Eagle’s Minimum Essential Medium
ESRD: End-stage renal disease
FBS: Fatal bovine serum
FITC: Fluorescein isothiocyanate
GFR: Glomerular filtration rate
LDH: Lactate dehydrogenase
MDCK: Madin-Darnby Canine Kidney
PI: Propidium iodide
T2D: Type 2 diabetes
